# The Effect of Osteopontin on Microglia

**DOI:** 10.1155/2017/1879437

**Published:** 2017-06-18

**Authors:** Huan Yu, Xiaohong Liu, Yisheng Zhong

**Affiliations:** Department of Ophthalmology, Ruijin Hospital Affiliated Medical School, Shanghai Jiaotong University, Shanghai 200025, China

## Abstract

Osteopontin (OPN) is a proinflammatory cytokine that can be secreted from many cells, including activated macrophages and T-lymphocytes, and is widely distributed in many tissues and cells. OPN, a key factor in tissue repairing and extracellular matrix remodeling after injury, is a constituent of the extracellular matrix of the central nervous system (CNS). Recently, the role of OPN in neurodegenerative diseases has gradually caused widespread concern. Microglia are resident macrophage-like immune cells in CNS and play a vital role in both physiological and pathological conditions, including restoring the integrity of the CNS and promoting the progression of neurodegenerative disorders. Microglia's major function is to maintain homeostasis and the normal function of the CNS, both during development and in response to CNS injury. Although the functional mechanism of OPN in CNS neurodegenerative diseases has yet to be fully elucidated, most studies suggest that OPN play a role in pathogenesis of neurodegenerative diseases or in neuroprotection by regulating the activation and function of microglia. Here, we summarize the functions of OPN on microglia in response to various stimulations in vitro and in vivo.

## 1. **Introduction**

Osteopontin (OPN) is a proinflammatory cytokine that can be secreted from many cells, including activated macrophages and T-lymphocytes, and is widely distributed in many tissues and cells [1]. OPN has been shown to be a constituent of the extracellular matrix of the central nervous system (CNS) [2, 3]. Recently, OPN has been studied in several physiological and pathological conditions where its production is upregulated in response to either inflammation or injury [2], especially in CNS. It has been reported that OPN play a role in neurodegenerative diseases such as multiple sclerosis (MS) [4, 5], Parkinson's disease (PD) [6, 7], and Alzheimer's disease (AD) [8, 9]. Microglia are the resident macrophage-like immune cells in CNS and play a vital role in both physiological and pathological conditions, including restoring the integrity of the CNS and promoting the progression of neurodegenerative disorders [10]. Under physiological conditions, most microglia remain in a resting state. In a variety of pathological conditions of CNS, such as brain trauma [11], cerebral ischemia [12], infection [13], and degenerative diseases [14], microglia can rapidly participate in the pathophysiology of brain damage via its activation, proliferation, migration, phagocytosis, and expression of inducible nitric oxide synthase (iNOS), nitric oxide (NO), and a number of proinflammatory cytokines [15]. Based on the effect of OPN and microglia reported recently, most studies suggest that OPN play a role in pathogenesis of neurodegenerative diseases or in neuroprotection by regulating the activation and function of microglia [16–18]. Thus, we will sum up the effect of OPN on microglia in several aspects including proliferation, migration, phagocytosis, and expression of proinflammatory cytokines.

## 2. The Characteristics of OPN

Osteopontin (OPN) is a highly negatively charged phosphoglycoprotein, which can be synthesized and secreted by different kinds of cells, including osteoblasts, fibroblasts, epithelial cells, vascular smooth muscle cells, a variety of tumor cells, activated T cells, and macrophages [1, 19–23]. It is widely distributed in many tissues like bone, kidney, muscle, and bladder and is also found in biological fluids, such as milk, urine, blood, and seminal fluids [24].

OPN is expressed by a single-copy gene with a 34-kDa nascent protein composed of 300 amino acid residues. The human gene contains 7 exons and maps to the long arm of chromosome 4 (4q21–23) [25], whereas, in a mouse, the gene is situated at chromosome 5 locus of the Rickettsia Resistance Gene while a pig gene is on chromosome 8. The molecular weight of OPN is between 44 KD and 66 KD, depending on the particular species and the type of cell [26], of which aspartic acid, serine, and glutamic acid residues account for a higher proportion. OPN has a specific amino acid sequence (Arg-Gly-Asp) and is also termed RGD-containing protein, which is a unique structure in the protein that mediates cell attachment [24]. There are two subtypes of OPN, the secretory OPN (sOPN) and the intracellular OPN (iOPN) [27]. The sOPN works by binding to the extracellular receptors expressed by the target cells while the iOPN acts by binding to MyD88, which is located in the downstream of the toll-like receptor. In vivo, both kinds of OPN can be involved in the immune regulation process through different pathways. OPN receptors include integrins and CD44 families, mainly distribute in astrocytes, osteoclasts, T cells, vascular smooth muscle cells, and the surface of macrophages [28]. OPN bind to the receptors to promote cell chemotaxis, adhesion, and migration and participate in bone resorption, inflammation, and immune processes [1].

As mentioned earlier, OPN is a constituent of extracellular matrix of normal CNS, playing a key role in tissue repairment and extracellular matrix remodeling after an injury. More recently, the role of OPN in neurodegenerative diseases has gradually attracted people's attention.

## 3. The Characteristics of Microglia

The CNS consists of neurons and glial cells, with the quantity of glial cells being ten times the amount of neurons [29]. Glial cells include macroglia and microglia, and the microglia account for 5% to 20% of the total number of glial cells [29, 30], equal to the number of neurons [31, 32]. Microglia, widely spread in all brain regions are cells of the mononuclear-phagocyte lineage [33]. As the resident immune effective cells of the CNS, microglia mediate immune-related processes [34]. Under physiological conditions, most microglia remain in a resting state, with ramified processes constantly retracting from the surrounding neural tissues. During a pathological stimulation the microglia get rapidly activated in response to even minor pathological changes in the CNS, becoming the earliest reaction cells after a CNS injury [11, 12, 35]. This immune function and wild distribution enable microglia to play an important role in maintaining homeostasis and repair the damaged CNS [31].

Recent studies have revealed the regional microglial diversity and heterogeneity [36, 37]. Grabert et al. performed the first genome-wide analysis of microglia from discrete brain regions across the adult lifespan (at three different ages) of the mouse. Their study revealed microglia as richly diverse cells under steady-state conditions and that microglial aging occurs nonuniformly in a region-dependent manner. They indicated that augmentation of the distinct cerebellar immunophenotype and a contrasting loss in distinction of the hippocampal phenotype among forebrain regions were key features during aging [37]. These findings may explain why neurodegeneration often occurs in disease-specific spatially restricted patterns.

Microglia are the main cells involved in the immune and inflammatory reactions in the neurodegenerative diseases. It is widely accepted that activated microglia exert dual functions, that is, proinflammatory (M1) and anti-inflammatory (M2) functions [38]. The direction of the polarization depends on their exposure to the cytokine byproducts of polarized T cell subsets (Th1: IFN*γ* or Th2: IL-4). In this theory, M1 phenotypic cells hinder CNS repair and expand tissue damage by producing destructive proinflammatory mediators. By contrast, M2 phenotypic cells promote brain recovery by clearing cell debris, resolving local inflammation, and releasing a plethora of trophic factors. The in vivo status of activated microglia is probably on a continuum between these two extreme states, which means that microglia can be polarized into an activation state that is intermediate between a neuroharmful and a protective state [39]. However, in recent years, some emerging views have been raised. Martínez and Gordon put forward that the long-held M1/M2 convention for describing macrophage polarization may be more applicable to in vitro systems than for far more complex in vivo environments, as mixed phenotypes are commonly seen [40]. The latest point of Ransohoff was the lack of predicted transcriptional organization found between polarization states induced in several disease models as demonstrated by ex vivo expression profiling of microglia, indicating that microglial reactivity is multifactorial and injury-specific, thus, unlikely even to fall along a linear continuum. The application of M1/M2 markers for the in vivo description of microglia activation states is inadequate in defining the injury-resolving capacity of these cells [41]. Thus, attempting to classify the proinflammatory phenotype of aged microglia as M1 may be too simplistic in that it ignores the adaptive requirement of these cells to respond to the demands of a changing microenvironment over the lifespan [42]. In recent years, the senescence-associated secretory phenotype (SASP) has been utilized to more accurately describe aged senescent cells [43]. Although SASP criteria have yet to be established specifically for microglia, emerging studies suggest a framework for one will emerge in the next few years [44].

## 4. The Effect of OPN on Microglia

### 4.1. OPN Is Mainly Synthesized and Secreted by Microglia under Stress Conditions

A large amount of experiments in vitro or vivo has shown that OPN expression was significantly increased after cerebral ischemia [2, 45–48]. Ellison et al. found that, in a rat model of Middle Cerebral Artery Occlusion (MCAO), the level of OPN mRNA and protein began to increase in twelve hours after MCAO, reaching a peak in five days, which was 49.5 times higher than that of the control group. Within 48 hours, after the onset of MCAO, OPN mRNA appeared mainly in the surrounding area of infarction, and after five days, there was a noticeable increase in the number of OPN mRNA in the infarction core, disappearing in the surrounding area [45]. Shin et al. showed that activated microglia and macrophages were the main source of OPN. With the increase of OPN, the expression of CD44 receptor and integrin receptor *α*_V_*β*_3_ increased in the ischemic brain. OPN combined with its receptors to promote the activation and migration of glia, resulting in the formation of a glial scar and the tissue repairing process following ischemic injury [49].

Similar findings were found in the studies of spinal cord and peripheral nerve injury. There was only a small amount of OPN expressed in normal spinal cord, while, in a variety of spinal cord injury (SCI) model, OPN expression was significantly upregulated. OPN mRNA was upregulated in 24 hours and peaked in three days after a crash injury, and the level of OPN mRNA was seven times higher than that of the control group [50]. Hashimoto et al. found the upregulation of OPN expression in activated microglia/macrophages and astrocytes by in situ hybridization. In the normal spinal cord, OPN mRNA was detected at a low level only in a subset of spinal motoneurons but dramatically increased following avulsion in activated microglia/macrophages and astrocytes. Therefore, he proposed that upregulation of OPN after spinal root avulsion is involved in the protection of neurons and the posttraumatic inflammatory response in microglia/macrophages and astrocytes. In contrast, the neurons, which could not express enough OPN, would be selected to degenerate and die [51].

Iczkiewicz and coworkers demonstrated that OPN protein expression is decreased in surviving dopaminergic neurons in Parkinson's disease (PD) and is present in activated microglia [52]. Several researches in Alzheimer's disease (AD) in both animals and humans also revealed this relationship between OPN and microglia. OPN has been shown to be the most strongly upregulated cytokine in activated microglia following hippocampal kainic acid injection in the Senescence-accelerated mouse prone 10 (SAMP10), a mouse strain characterized by accelerating senescence and early cognitive decline [53]. These findings have not only revealed the source of OPN but also suggested that OPN is involved in neurodegenerative diseases.

### 4.2. OPN Increases Microglia Survival under Stress Conditions

Rabenstein et al. cultured microglial cells in serum-free medium for 48 hours and treated them with different concentrations of OPN. They found that the number of microglial cells in the test group was 100-fold higher than that of the control. And the dead microglia visibly decreased with treatment of 6.25 *μ*g/ml or 12.5 *μ*g/ml OPN, which indicated that the appropriate concentration of OPN could increase the survival of microglia under stress conditions [54]. And they speculated that OPN might enhance microglia survival under the stress of nutrient deprivation after cerebral ischemia, which supports the notion that OPN serves as an important regulatory protein of neuroinflammation.

### 4.3. The Effect of OPN on Proliferation of Microglia

It has been confirmed that OPN can stimulate the proliferation of epithelial cells [55]; moreover, OPN has been associated with tumor proliferation [56]. Yet, its effect on microglia proliferation is still controversial. Tambuyzer et al. found that the proliferation of microglial significantly increased compared with control cells when a lower concentration (10 fM) recombinant OPN was added to the culture medium, which indicated that OPN also stimulated proliferation of microglia [18]. However, Rabenstein et al. put forward the opposite conclusion. They analyzed the expression of the proliferation marker Ki67 on the mRNA level. Ki67-mRNA was quantitatively assessed after OPN treatment using real-time quantitative polymerase chain reaction (RT-qPCR). Microglia treated with OPN at 6.25 *μ*g/ml for 24 hours or 72 hours did not contain more Ki67-mRNA than untreated microglia, suggesting that OPN had no effect on microglia proliferation [54].

### 4.4. The Effect of OPN on Phagocytosis of Microglia

At present, it is still controversial whether OPN affects the phagocytic activity of microglia. In the experiment of Rabenstein et al., microglia were treated with OPN at 6.25 or 12.5 *μ*g/ml for 24 hours and then zymosan microbeads were added for 2 hours to subsequently quantify phagocytic activity photometrically. The amount of microbeads phagocytosed by microglia was unaffected by OPN treatment compared to untreated control cells, suggesting the phagocytic activity of microglia did not affected by OPN [54], while Tambuyzer et al. proposed that freshly harvested microglia initially had a high phagocytic activity, on which OPN treatment had no significant effect. After 24 hours of culture in the DMEM complete medium, their phagocytic activity was reduced to 40% of this initial level. Treating these cells with OPN for 24 hours significantly increased the phagocytotic activity compared with microglia cultured in control medium. The uptake of beads by microglia treated with 1 nM OPN was almost doubled that of control cells, thereby largely restoring the activity to the level observed immediately after harvesting [18]. Therefore, they believe that OPN can increase the phagocytic activity of microglia. Besides, OPN has been correlated with increased phagocytosis by brain macrophages in a rat stroke model [49] and also by peripheral monocytes/macrophages [57].

### 4.5. The Effect of OPN on Migration of Microglia

Zohar et al. reported that OPN induced phosphorylation of adhesion kinase (FAK) in microglia by RGD binding to integrin receptors, which subsequently activated Ras and mitogen-activated protein kinase (MAPK) via Grb2/SOS or FAK/Src and regulated cytoskeleton protein assembly and cell migration. Intracellular OPN and hyaluronic acid-CD44-ERM combined into a complex change the cytoskeleton to promote cell movement [58]. However, Rabenstein et al. found that OPN did not affect microglia migration by using a modified Boyden chamber transwell migration assay [54].

### 4.6. OPN Inhibits Microglial Superoxide Production

Microglia cells treated with recombinant OPN and subsequently stimulated with PMA showed a significant inhibition of superoxide production. This occurred at lower OPN concentrations (10 fM) when the microglia cells were grown in the absence of foetal bovine serum. With serum present, microglial superoxide production was significantly inhibited only at a higher OPN concentration (10 pM) [18]. These experiments could mimic the normal CNS environment (serum-free) and its disturbance during neuropathology with blood-brain barrier disruption (with serum). This finding supports the notion that OPN may have neuroprotective properties during stroke [59, 60].

Rabenstein et al. found that LPS-stimulation led to a significant increase in iNOS-positive cell count. When 1 ng/ml LPS-stimulated microglia were cotreated with 12.5 *μ*g/ml OPN, the number of iNOS-positive cells decreased significantly. However, after stimulation with LPS at 10 mg/ml, cotreatment with OPN did not reduce the number of iNOS-positive cells. The same result was reflected in the research about NO, suggesting a dose-dependent effect of OPN on LPS-induced NO-release from primary microglia [54].

Wolak and his colleagues noted that the lack of OPN increased NADPH-oxidase protein expression and therefore increased oxidative stress [61]. OPN has been identified as an “oxidative stress-sensitive cytokine” upregulated by oxidative stress [62, 63]. Therefore, it is conceivable that OPN, as an oxidative stress-regulated protein, provides a negative feedback for oxidative metabolism in inflammatory cells or could even directly scavenge oxygen radicals [64, 65].

### 4.7. OPN Modulates Release of Proinflammatory Cytokines

In the experiments of Rabenstein et al., LPS-stimulated microglia were then cotreated with 6.25 or 12.5 *μ*g/ml OPN, respectively. Compared to untreated cells stimulated with LPS, IL-6-release was significantly reduced after cotreatment with OPN at a concentration of 12.5 *μ*g/ml. However, the lower concentration of 6.25 *μ*g/ml OPN did not reduce IL-6-release. The experiments of TNF-*α* release displayed the same results. Thus, there is also a dose-dependent effect of OPN on LPS-induced IL-6/TNF-*α*-release from microglia [54]. It is worth mentioning that although this result is obtained without interference from blood-derived macrophages, Patouraux et al. also reported, in a macrophage cell line, downregulation of OPN enhancing iNOS expression and leading to an upregulation of iNOS, TNF-*α*, and IL-6 in response to lipopolysaccharides [66].

There are large quantities of experiments in vitro, which show that OPN can promote the survival of microglia under stress conditions and have an anti-inflammatory effect in mild to moderate inflammatory environments. These studies support the concept that OPN is an important regulator of neuron-inflammation [67, 68]. It can regulate the activity of microglia and promote cell regeneration after a stroke to the brain [18]. However, as the experiments in vitro cannot completely simulate the pathological environment of CNS, the effects and influences of OPN on microglia remain to be further explored and verified in animal experiments.

According to the present researches in the neurodegenerative disease such as PD and AD, some scientists supposed that OPN play a role in anti-inflammatory and antiapoptotic properties and regulating iNOS transcription, reactive oxygen species production, and cytokines levels, which are expressed by activated microglia [15, 69–71].

## 5. Conclusion

More and more scholars believe that OPN is likely to be an effective therapeutic target for neurodegenerative diseases [72]. There are also a large number of researches, which show that OPN may be involved in the pathogenesis and neuroprotective process of neurodegenerative diseases by modulating the activation and function of microglia [17, 73]. However, in order to exclude the indirect effects and epiphenomena that complicate in vivo studies, most researches were carried out in vitro. Besides, the specific mechanism of OPN has not been fully elucidated. Therefore, more in vivo studies and in-depth exploration of the role of OPN in the development of neurodegenerative diseases is required, especially in its impact on microglia, which will not only be beneficial in explaining the pathogenesis of neurodegenerative diseases but also contribute to the clinical screening and prognosis judgment of these diseases, providing new ideas for the development of therapeutic drugs.
